# Virion endocytosis is a major target for murid herpesvirus-4 neutralization

**DOI:** 10.1099/vir.0.040790-0

**Published:** 2012-06

**Authors:** Daniel L. Glauser, Laurent Gillet, Philip G. Stevenson

**Affiliations:** 1 Division of Virology, Department of Pathology, University of Cambridge, UK; 2 Immunology–Vaccinology, Faculty of Veterinary Medicine, University of Liège, Liège, Belgium

## Abstract

Herpesviruses consistently transmit from immunocompetent carriers, implying that their neutralization is hard to achieve. Murid herpesvirus-4 (MuHV-4) exploits host IgG Fc receptors to bypass blocks to cell binding, and pH-dependent protein conformation changes to unveil its fusion machinery only after endocytosis. Nevertheless, neutralization remains possible by targeting the virion glycoprotein H (gH)–gL heterodimer, and the neutralizing antibody responses of MuHV-4 carriers are improved by boosting with recombinant gH–gL. We analysed here how gH–gL-directed neutralization works. The MuHV-4 gH–gL binds to heparan sulfate. However, most gH–gL-specific neutralizing antibodies did not block this interaction; neither did they act directly on fusion. Instead, they blocked virion endocytosis and transport to the late endosomes, where membrane fusion normally occurs. The poor endocytosis of gH–gL-neutralized virions was recapitulated precisely by virions genetically lacking gL. Therefore, driving virion uptake appears to be an important function of gH–gL that provides a major target for antibody-mediated neutralization.

## Introduction

Gammaherpesvirus neutralization by immune sera and mAbs is well-described (Thorley-Lawson & Poodry, 1982; Stevenson & Doherty, 1998; Dialyna *et al.*, 2004). However, the persistent infectivity of herpesvirus carriers and the limited evidence for immune selection of viral antigenic variants imply that *in vivo* neutralization is harder to achieve. Consistent with such an idea, immunization with the Epstein–Barr virus (EBV) gp350, which is a potent target for neutralization *in vitro* (Thorley-Lawson & Poodry, 1982), failed to reduce infection rates *in vivo* (Sokal *et al.*, 2007).

Narrow species tropisms limit analyses of EBV and Kaposi’s sarcoma-associated herpesvirus (KSHV). Murid herpesvirus-4 (MuHV-4) (Barton *et al.*, 2011; Stevenson *et al.*, 2009) consequently provides a useful tool for exploring how gammaherpesviruses and antibody interact. Antibody reduces MuHV-4 lytic spread *in vivo* (Kim *et al.*, 2002; Gangappa *et al.*, 2002). However, this reflects IgG Fc receptor (FcR) engagement rather than neutralization (Wright *et al.*, 2009). Immune sera potently neutralize fibroblast infection by MuHV-4 *in vitro*, but they neutralize host entry much less well (Gillet *et al.*, 2007a), possibly because ‘neutralized’ virions can use opsonization pathways such as FcRs to infect when normal cell binding is blocked (Rosa *et al.*, 2007). Thus, robust neutralization requires a block to cell penetration rather than just cell binding, and this seems to be more difficult to achieve.

Although MuHV-4-immune sera do not block host entry efficiently, they can be effective at high dose (Gillet *et al.*, 2007a). This probably reflects antibody binding to the glycoprotein H–glycoprotein L heterodimer (gH–gL), as this is the target of most neutralizing mAbs recovered from virus carriers (Gill *et al.*, 2006). gH–gL is poorly immunogenic in the context of whole virus (Gillet *et al.*, 2007b). However, gH–gL-specific antibodies can be boosted with recombinant protein, and this improves the capacity of immune sera to block entry (Gillet *et al.*, 2007a). In contrast, few mice make neutralizing responses to gB, even after boosting (May & Stevenson, 2010). gH–gL also provides a neutralization target for EBV (Chesnokova & Hutt-Fletcher, 2011) and KSHV (Naranatt *et al.*, 2002). An important unanswered question is how this neutralization works. The MuHV-4 gH–gL, like that of KSHV (Hahn *et al.*, 2009), binds to heparan sulfate (HS) (Gillet *et al.*, 2008a), and HS binding is important for MuHV-4 to infect (de Lima *et al.*, 2004). However, the virion gp70 also fulfils this role (Gillet *et al.*, 2007c) and gL^−^ virions show only a modest cell-binding defect (Gillet *et al.*, 2007d); they show a much greater post-binding infection defect (Gillet *et al.*, 2008b). The implication is that neutralizing antibodies target a downstream gH–gL function.

gH plays an essential, conserved role in herpesvirus membrane fusion (Forrester *et al.*, 1992). It binds to gL (Hutchinson *et al.*, 1992), and gH–gL-specific antibodies block membrane fusion by both herpes simplex virus (HSV) (Fuller *et al.*, 1989) and EBV (Miller & Hutt-Fletcher, 1988). However, whether the block is direct is unclear. MuHV-4 infection is endocytic and, after endocytosis, gH changes antigenically from gH–gL to ‘gH-only’ (Gillet *et al.*, 2008b). Virions lacking gL remain infectious (Gillet *et al.*, 2007d), implying that gH engages in fusion as gH-only rather than as gH–gL, and antibodies specific for gH-only neutralize gL^−^ MuHV-4 (Gillet *et al.*, 2009), consistent with it being functionally important. They do not neutralize gL^+^ MuHV-4 because such virions express little gH-only until after endocytosis. Thus, the gH–gL-directed neutralization of gL^+^ virions may act upstream of the fusion reaction.

In this study, we aimed to understand how the MuHV-4 gH–gL provides a neutralization target when its epitopes are normally lost pre-fusion. In contrast to gB-neutralized virions, which are arrested at the point of fusion in late endosomes, gH–gL-neutralized virions failed to reach this site. Both gH–gL-neutralized and gL-deficient virions were endocytosed poorly, suggesting that gH–gL-driven endocytosis is an important target for virion neutralization.

## Results

### gH–gL-directed neutralization can act in different ways

**Fig. 1.**
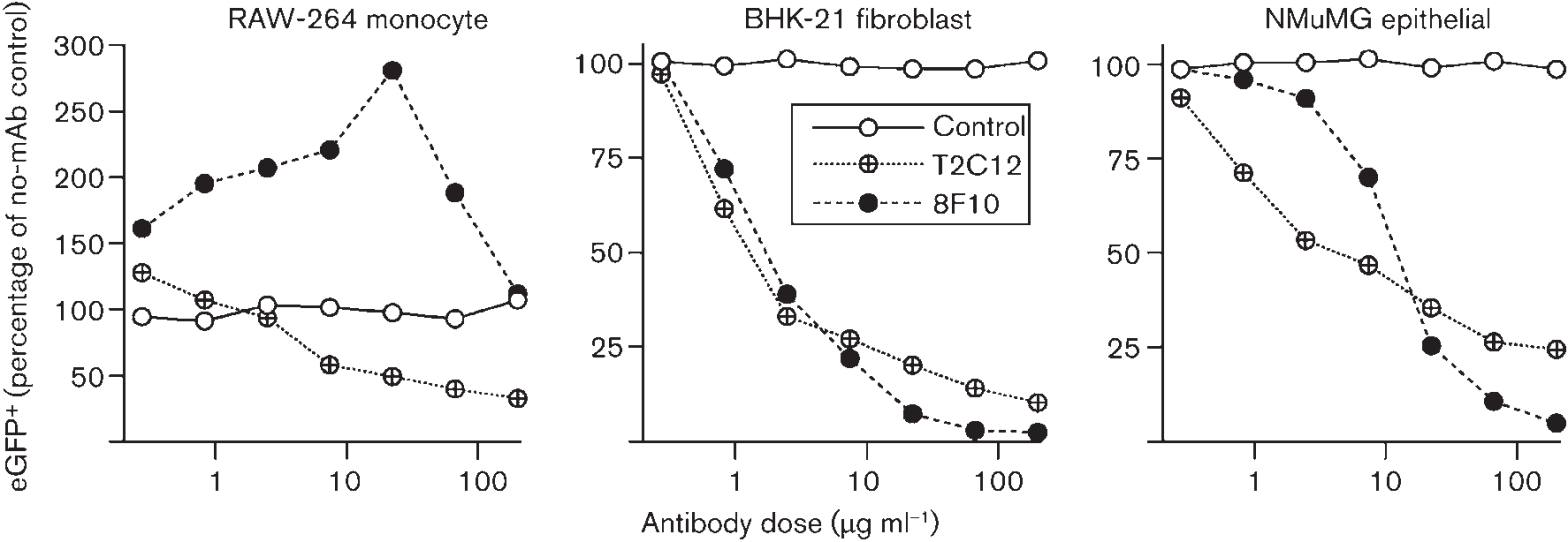
Comparison of virion neutralization by gH–gL-specific mAbs T2C12 and 8F10. MuHV-4 virions expressing eGFP from an intergenic EF1α promoter were incubated (2 h, 37 °C) with antibody dilutions, then divided between RAW-264 monocytes (3 p.f.u. per cell), BHK-21 fibroblasts (0.5 p.f.u. per cell) and NMuMG epithelial cells (0.5 p.f.u. per cell). After overnight incubation (37 °C) in the presence of 100 µg phosphonoacetic acid ml^−1^ to prevent secondary spread, the cells were analysed for eGFP expression by flow cytometry. The MHC class I-specific IgG_2a_ mAb 28.14.8 provided a control.

mAb 8F10 (IgG_2a_) is representative of the minority of gH–gL-specific neutralizing mAbs that block gH–gL binding to HS (four of 13 mAbs from three different fusions); mAb T2C12 (IgG_2a_) is representative of the majority (nine of 13 mAbs) that do not block HS binding (Gillet *et al.*, 2008a). Blocking HS binding with immune serum inhibits fibroblast and epithelial-cell infections by MuHV-4, but enhances macrophage infection because antibody-coated virions can bind to and infect via FcRs (Rosa *et al.*, 2007). mAb 8F10 similarly inhibited BHK-21 (FcR^−^ fibroblast) and NMuMG (FcR^−^ epithelial) infections, but increased RAW-264 cell infection (FcR^+^ monocyte) (Fig. 1), implying that it mainly blocked cell binding. T2C12, in contrast, inhibited both FcR^−^ and FcR^+^ infections, consistent with a block downstream of cell binding (Gill *et al.*, 2006).

### Tracking virion entry by immunofluorescence

**Fig. 2.**
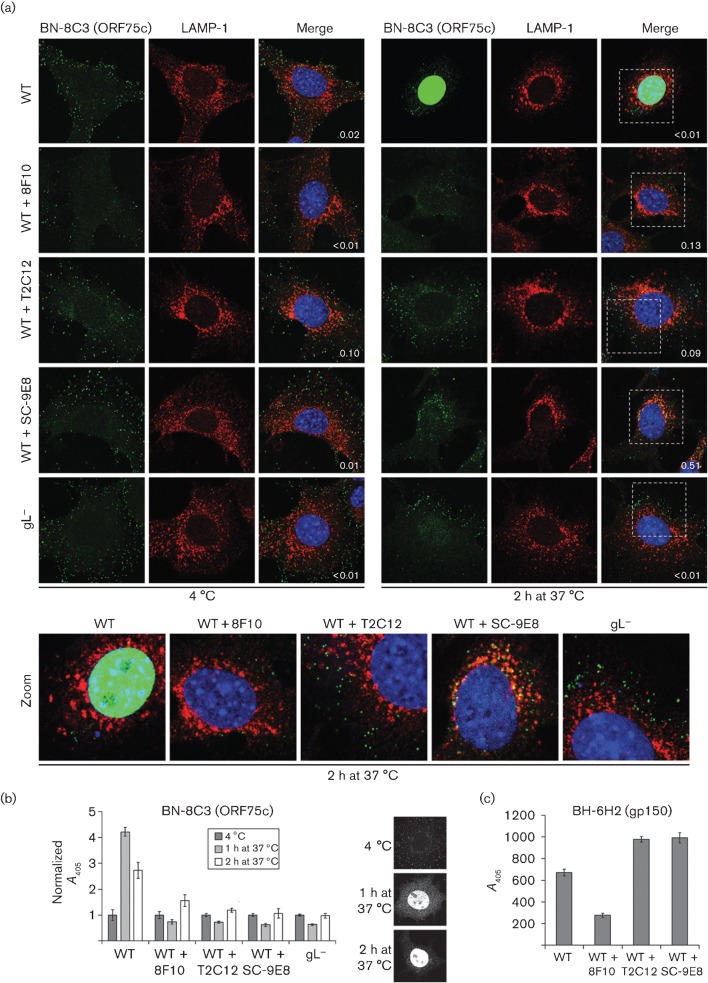
Transport of neutralized virions. (a) Wild-type (WT) MuHV-4 virions (3 p.f.u. per cell) were left untreated or pre-incubated (2 h, 37 °C) with mAbs 8F10 (anti-gH–gL, IgG_2a_), T2C12 (anti-gH–gL, IgG_2a_) or SC-9E8 (anti-gB, IgG_2a_) (400 µg ml^−1^) before binding to NMuMG cells (2 h, 4 °C). For comparison, other NMuMG cells were similarly exposed to gL^−^ virions (50 p.f.u. per cell, so as to get equivalent binding). The cells were then washed with PBS and either fixed immediately or first further incubated (2 h, 37 °C) to allow virion endocytosis. The cells were then stained for the ORF75c virion tegument protein with mAb BN-8C3 (IgG_1_, green) and for the late endosomal marker LAMP-1 (red), and with DAPI (blue). Red/green co-localization appears as yellow. Equivalent data were obtained in a repeat experiment. In this and all subsequent figures, the data shown are fully representative of at least 100 cells examined. The confocal settings were the same for the corresponding images at 4 °C and after 2 h at 37 °C. The numbers give the fraction of green signal co-localizing with red signal. The zoomed images show in more detail the relationship between virions (green) and endosomes (red). (b) As in (a), cells were exposed to virions for 2 h at 4 °C, washed in PBS, then either analysed immediately or first incubated for 1 or 2 h at 37 °C, but antibody binding was detected with an IgG_1_-specific alkaline phosphatase-conjugated secondary antibody and incubation with *p*-nitrophenylphosphate substrate, and quantified by measuring *A*_405_. For each condition, the *A*_405_ was normalized to the value obtained at 4 °C. The bars show mean±sem values from six wells. The ORF75c signal after incubation at 37 °C was significantly higher for non-neutralized WT virions than for gL^−^ or neutralized WT virions (*P*<0.008 by Student’s *t*-test). The images show the distribution of ORF75c after virion binding at 4 °C, and after incubation at 37 °C for 1 or 2 h. (c) In a similar experiment to (b), virions were bound to cells for 2 h at 4 °C and detected with the gp150-specific IgG_2b_ mAb BH-6H2 plus an alkaline phosphatase-conjugated IgG_2b_-specific secondary antibody. The bars show mean±sem *A*_405_ values from six wells. The signal with 8F10-neutralized virions was reduced significantly relative to other treatments (*P*<10^−5^). Equivalent data were obtained in a repeat experiment.

To investigate how gH–gL-directed neutralization inhibited MuHV-4 entry into NMuMG epithelial cells, we visualized virions by isotype-specific immunofluorescence for the ORF75c tegument protein (Fig. 2a). This allowed us to track both cell binding and membrane fusion, as ORF75c relocates rapidly to the cell nucleus when released by fusion (Gaspar *et al.*, 2008). After incubation at 4 °C, ORF75c remained in virions bound to the plasma membrane; after 1 h at 37 °C, it was both in the cytoplasm – either in endocytosed virions or just released from them – and in the nucleus (Fig. 2b); after 2 h at 37 °C it was almost entirely in the nucleus. ORF75c staining increased during its relocation, presumably because release from the virion tegument made it more accessible to antibody. This increase was evident both by immunofluorescence of individual cells (Fig. 2a) and by ELISA of virus-exposed cell populations (Fig. 2b). The ELISA signal was consistently stronger after 1 h at 37 °C than after 2 h (Fig. 2b), suggesting that ORF75c is either masked or degraded with time.

The number of virions binding to cells exceeded the number of p.f.u. added by as much as 100-fold. This was not surprising: even very lytic viruses often have particle/p.f.u. ratios of 10 or more, and MuHV-4 is far from uniformly lytic. An MuHV-4 mutant engineered for greater lytic infection (May *et al.*, 2004) showed 10-fold fewer virions binding per p.f.u., and was similar in particle/p.f.u. ratio to HSV-1 (Fig. S1, available in JGV Online). In addition to MuHV-4 not being uniformly lytic, it is possible that, in large transformed cells, the tegument of just one virion is insufficient to initiate infection. Virion tegument proteins play an important role in combating cellular defences such as the promyelocytic leukaemia (PML) protein (Everett & Chelbi-Alix, 2007), and this is one action of ORF75c (Gaspar *et al.*, 2008). An important point is that almost all of the wild-type virions bound to cells reached late endosomes and released their ORF75c after further incubation at 37 °C (Fig. 2a); therefore, there was no evidence of the bound virions being structurally defective.

### gH–gL-directed neutralization arrests virions downstream of cell binding

Only mAb 8F10 reduced the number of virions binding at 4 °C (Fig. 2a). However, both 8F10 and T2C12 blocked ORF75c transport to the nucleus (Fig. 2a) and its increased accessibility (Fig. 2b), as did the gB-specific mAb SC-9E8, which blocks membrane fusion (Glauser *et al.*, 2011). ELISA for the external virion component gp150 with mAb BH-6H2 (Fig. 2c) confirmed that 8F10, but not T2C12 or SC-9E8, reduced cell binding.

### Incoming T2C12-neutralized and gL^−^ virions both remain distributed peripherally

Although both T2C12 and SC-9E8 allowed cell binding, they caused different blocks, as only SC-9E8 allowed virions to reach late endosomes and co-localize with LAMP-1 (Fig. 2a, zoomed images); T2C12-neutralized virions remained peripheral to late endosomes. The same peripheral distribution was seen with gL^−^ virions, whereas the endosomal virion arrest by SC-9E8 matched that observed with inhibitors of endosomal acidification such as bafilomycin and concanamycin A (Glauser *et al.*, 2011).

**Fig. 3.**
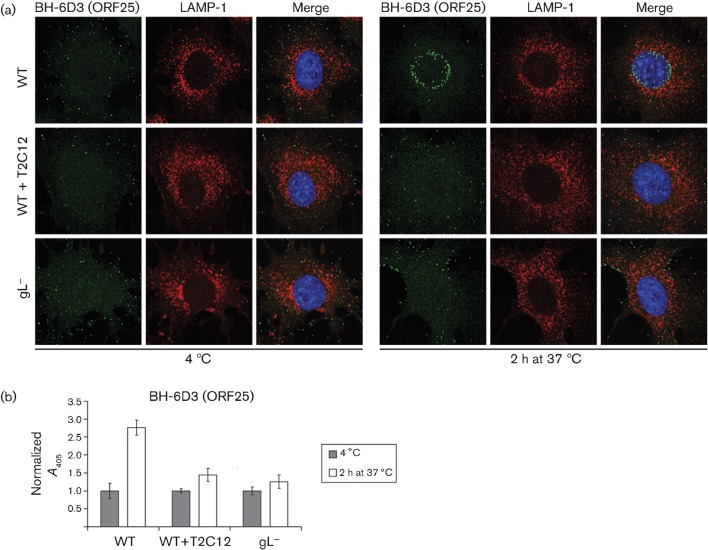
Capsid migration of T2C12-neutralized and gL^−^ virions. (a) WT MuHV-4 virions (3 p.f.u. per cell) were left untreated or pre-incubated (2 h, 37 °C) with mAb T2C12 (anti-gH–gL, IgG_2a_, 400 µg ml^−1^). WT (3 p.f.u. per cell) and gL^−^ (50 p.f.u. per cell to give equivalent binding) virions were then bound to NMuMG cells for 2 h at 4 °C. The cells were washed with PBS and either fixed immediately or first incubated (2 h, 37 °C) to allow virion endocytosis. All cells were then stained for the ORF25 virion capsid component with mAb BH-6D3 (IgG_1_, green), for the late endosomal marker LAMP-1 (red), and with DAPI (blue). Equivalent data were obtained in a repeat experiment. (b) Cells were exposed to WT virions with or without T2C12 neutralization or to gL^−^ virions as in (a), but antibody binding was detected with an alkaline phosphatase-conjugated IgG_1_-specific secondary antibody, *p*-nitrophenylphosphate substrate and *A*_405_. For each condition, the *A*_405_ was normalized to the value obtained at 4 °C. The bars show mean±sem values from six wells. After incubation at 37 °C, the non-neutralized WT signal was significantly higher than that of gL^−^ or T2C12-treated WT virions (*P*<0.0005 by Student’s *t*-test). Equivalent data were obtained in two further experiments.

Peripheral virion arrest by mAb T2C12 was confirmed by staining for the ORF25 capsid component with mAb BH-6D3 (Fig. 3a). After incubation at 37 °C, the capsids of wild-type virions migrated to the nuclear margin, consistent with their release by membrane fusion. In contrast, the capsids of T2C12-neutralized and gL^−^ virions remained outside late endosomes. Non-neutralized wild-type virions also showed an increase in ORF25 signal after incubation at 37 °C, consistent with released capsid making ORF25 epitopes more accessible (Fig. 3b), whereas T2C12-neutralized and gL^−^ virions showed little such increase.

**Fig. 4.**
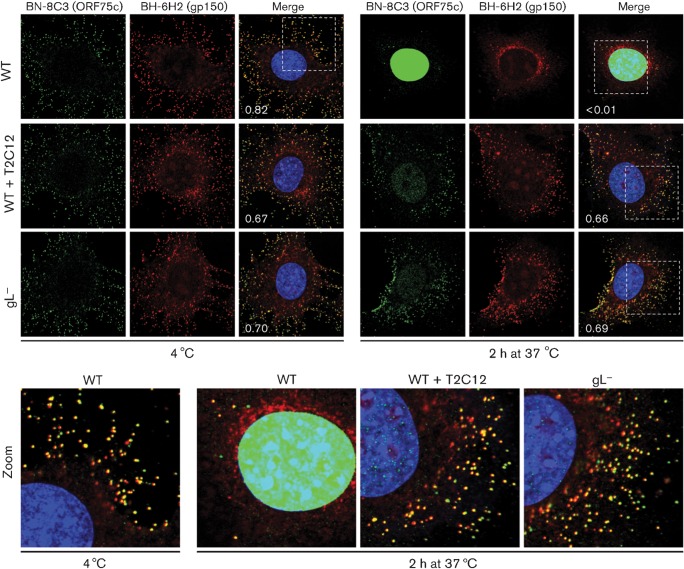
Monitoring virion entry by envelope/tegument co-localization. WT MuHV-4 virions (3 p.f.u. per cell) were left untreated or pre-incubated (2 h, 37 °C) with mAb T2C12 (anti-gH–gL, IgG_2a_, 400 µg ml^−1^). WT (3 p.f.u. per cell) and gL^−^ (50 p.f.u. per cell to give equivalent binding) virions were then bound to NMuMG cells for 2 h at 4 °C. The cells were washed with PBS and either fixed immediately or first incubated (2 h, 37 °C) to allow virion endocytosis. They were then stained for the ORF75c tegument component with mAb BN-8C3 (IgG_1_, green), for the gp150 envelope protein with mAb BH-6H2 (IgG_2b_, red) and with DAPI (blue). The numbers give the fraction of green signal co-localizing with red signal. The zoomed images show this relationship in more detail.

We also used gp150/ORF75c co-localization to track virion entry (Fig. 4). After binding at 4 °C, wild-type virions showed extensive gp150/ORF75c co-localization; after incubation at 37 °C, little co-localization remained. Thus most, if not all, wild-type virions underwent membrane fusion. In contrast, T2C12-neutralized and gL^−^ virions maintained extensive gp150/ORF75c co-localization, even after incubation at 37 °C.

### T2C12-neutralized and gL^−^ virions are endocytosed poorly

**Fig. 5.**
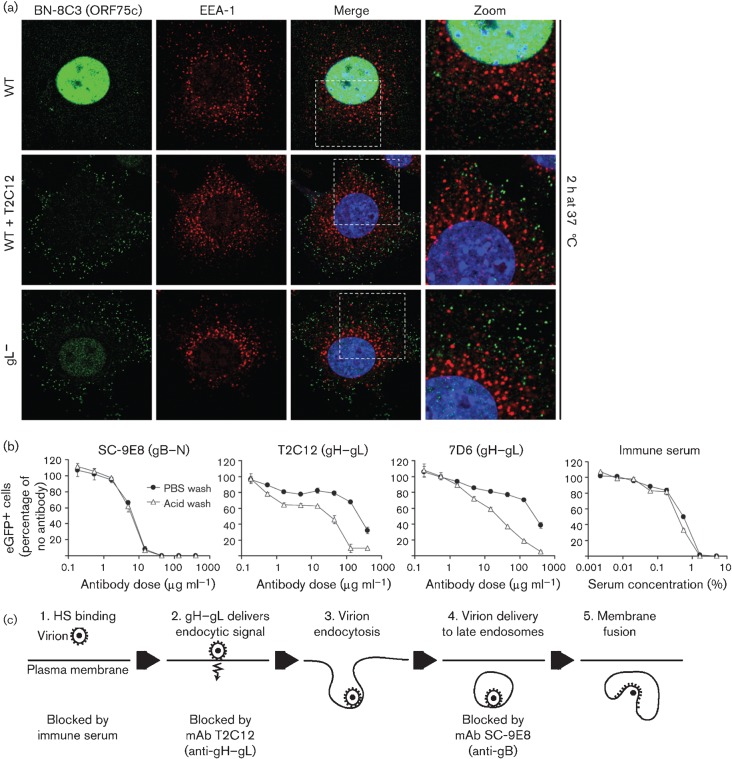
gH–gL-neutralized virions show defective endocytosis. **(**a) WT MuHV-4 virions (3 p.f.u. per cell) were left untreated or incubated (2 h, 37 °C) with mAb T2C12 (anti-gH–gL, IgG_2a_, 400 µg ml^−1^). These and gL^−^ virions (50 p.f.u. per cell to give equivalent binding) were then bound to NMuMG cells (2 h, 4 °C). The cells were washed with PBS and incubated (2 h, 37 °C) to allow virion endocytosis, washed, fixed, and stained for the ORF75c virion tegument protein with mAb BN-8C3 (IgG_1_, green), for the early endosomal marker EEA-1 (red) and with DAPI (blue). Significant co-localization (yellow) was not observed. The zoomed images show virion and endosome distributions in more detail. (b) eGFP-expressing MuHV-4 virions (0.2 p.f.u. per cell) were left untreated or incubated (2 h, 37 °C) with mAb SE-9E8 (anti-gB, blocks fusion), mAb T2C12 (anti-gH–gL), mAb 7D6 (anti-gH–gL) or MuHV-4 immune serum (blocks binding). The virus was then added to BHK-21 cells and incubated (2 h, 37 °C) to allow virus endocytosis. The cells were then washed with PBS to remove unbound virions or with pH 3 citrate/phosphate buffer to inactivate non-endocytosed virions, then incubated (16 h, 37 °C) in complete medium with 100 µg phosphonoacetic acid ml^−1^ and analysed for viral eGFP expression by flow cytometry. Each point shows the mean±sem of two experiments. Comparison across each dilution by Fisher’s exact test established that acid washing reduced infection significantly for mAbs T2C12 and 7D6 (*P*<0.02), but not for mAb SC-9E8 or for immune serum (*P*>0.5). (c) A schematic diagram shows where neutralization seems to act during MuHV-4 entry. Immune sera block virion binding to the plasma membrane; mAb T2C12 blocks the endocytosis of bound virions, and so presumably prevents the delivery of an endocytic signal by its target, gH–gL; gB-directed neutralization blocks membrane fusion and so strands virions in late endosomes.

T2C12-neutralized and gL^−^ virions not only failed to reach late endosomes, but also failed to reach the early endosomes marked by staining for early endosome antigen 1 (EEA-1) (Fig. 5a). Thus, they appeared not to be endocytosed. This was confirmed by analysing the susceptibility to PBS and acid washes of virions exposed to sub-neutralizing antibody concentrations (Fig. 5b). PBS wash removes unbound virions; acid wash also inactivates virions bound to the cell surface. SC-9E8-exposed virions incubated with cells for 2 h at 37 °C showed similar sensitivity to PBS and acid washes, consistent with a post-endocytic infection block. In contrast, virions exposed to T2C12 or to 7D6, another gH–gL-specific neutralizing mAb that does not block HS binding (Gillet *et al.*, 2008a), showed significantly greater sensitivity to acid wash. Thus, they were bound but not endocytosed. Immune sera mainly block cell binding (Gill *et al.*, 2006) and gave equal sensitivity to either wash. Immune sera have low gH–gL-specific antibody titres (Gillet *et al.*, 2007b) so, if cell binding is achieved, little further block to infection remains. Fig. 5(c) shows a schematic diagram of where we envisage that the different blocks occur.

### T2C12-neutralized virions maintain gB in its extracellular state

**Fig. 6.**
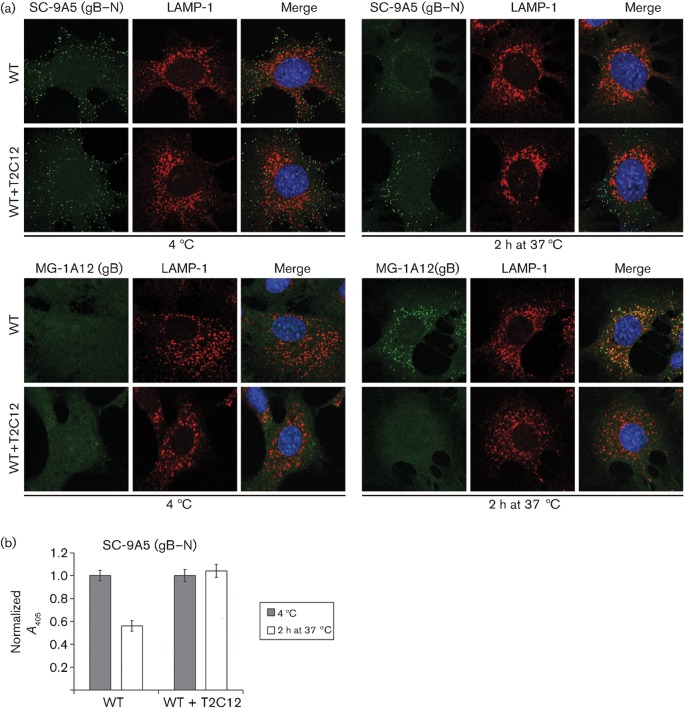
gH–gL-directed neutralization prevents conformation changes in gB. (a) WT MuHV-4 virions (3 p.f.u. per cell) were left untreated or pre-incubated (2 h, 37 °C) with mAb T2C12 (IgG_2a_, 400 µg ml^−1^), then bound to NMuMG cells (2 h, 4 °C). The cells were washed with PBS and either fixed immediately or first further incubated (2 h, 37 °C) to allow virion endocytosis, then stained with mAb SC-9A5 (IgG_3_), which is specific for pre-fusion gB, and with mAb MG-1A12 (IgG_2a_), which is specific for post-fusion gB (Glauser *et al.*, 2011). SC-9A5 was detected with an Alexa Fluor 488-conjugated goat anti-mouse IgG_3_ pAb; MG-1A12 was conjugated directly to Alexa Fluor 488 (both green). The cells were also stained for LAMP-1 (red) and with DAPI (blue). (b) NMuMG cells were exposed to untreated or T2C12-neutralized WT virions as in (a), then stained for pre-fusion gB with mAb SC-9A5. Antibody binding was detected with alkaline phosphatase-conjugated IgG_3_-specific secondary antibody, *p*-nitrophenylphosphate substrate and *A*_405_. For each condition, the *A*_405_ was normalized to the value obtained at 4 °C. The bars show mean±sem values from six wells. The non-neutralized WT signal was reduced significantly after incubation at 37 °C (*P*<10^−4^ by Student’s *t*-test), whereas the neutralized WT signal was not changed significantly (*P*>0.58). Equivalent data were obtained in a repeat experiment.

MuHV-4 virions show glycoprotein conformation changes both before membrane fusion and upon fusion itself. Notably, the gB epitope defined by mAb MG-1A12 is undetectable on extracellular virions, but revealed very early after endocytosis, even when fusion is blocked (Glauser *et al.*, 2012). The gB of T2C12-neutralized virions remained MG-1A12^−^ (Fig. 6a). It also retained the SC-9A5 epitope, which is normally present on extracellular virions and lost after endocytosis (Glauser *et al.*, 2011). SC-9A5 epitope retention in virion-exposed cell populations was also evident by ELISA (Fig. 6b). MG-1A12 ELISA was not possible because of antibody cross-reactivity with T2C12. Nonetheless, the lack of gB conformation changes supported the idea that mAb T2C12 blocked virion endocytosis.

**Fig. 7.**
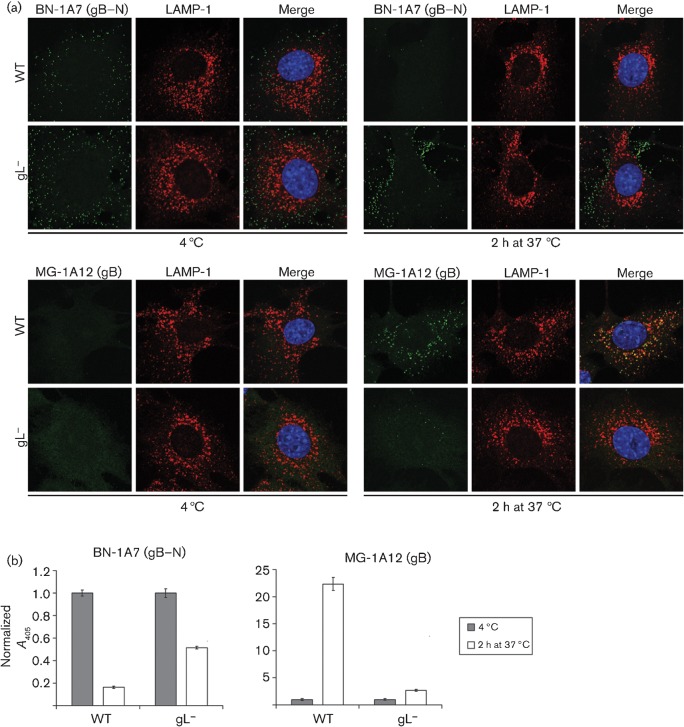
gL^−^ virions also show little conformation change in gB. (a) NMuMG cells were incubated (2 h, 4 °C) with WT (3 p.f.u. per cell) or gL^−^ (50 p.f.u. per cell for equivalent binding) MuHV-4, then washed with PBS and either fixed immediately or first further incubated (2 h, 37 °C) to allow virion endocytosis. The cells were stained for pre-fusion gB with mAb BN-1A7 (IgG_2a_) or for post-fusion gB with mAb MG-1A12 (IgG_2a_) (both green), for LAMP-1 (red) and with DAPI (blue). Co-localization appears as yellow. Equivalent data were obtained in three further experiments. (b) Cells and viruses were incubated as in (a), then antibody binding was detected with an alkaline phosphatase-conjugated IgG_2a_-specific secondary antibody, *p*-nitrophenylphosphate substrate and *A*_405_. For each condition, the *A*_405_ was normalized to the value obtained at 4 °C. The bars show mean±sem values from six wells. After incubation at 37 °C, the WT BN-1A7 signal was reduced significantly relative to gL^−^ (*P*<10^−9^ by Student’s *t*-test) and the WT MG-1A12 signal was increased significantly (*P*<10^−5^). Equivalent data were obtained in a repeat experiment.

gL^−^ virions (Fig. 7) similarly maintained gB in its ‘extracellular virion’ form (BN-1A7^+^MG-1A12^−^). Here, we used BN-1A7 (IgG_2a_) rather than SC-9A5 (IgG_3_) to detect pre-fusion gB because there was no need to allow for bound T2C12 (IgG_2a_); both epitopes are present on extracellular virions and lost after endocytosis (Glauser *et al.*, 2011). Immunofluorescence (Fig. 7a) showed that the BN-1A7 signal was maintained much better with gL^−^ than with wild-type virions. ELISA of gL^−^ virions (Fig. 7b) showed some loss of BN-1A7 detection at 37 °C, presumably because these virions bind less well, but the loss was substantially greater for wild-type virions. Both immunofluorescence (Fig. 7a) and ELISA (Fig. 7b) showed gL^−^ virions remaining largely MG-1A12^−^, while wild-type virions became strongly MG-1A12^+^. Tracking gB antigenic changes therefore supported the idea that having less functional gH–gL, through either mAb binding or gL disruption, impaired virion endocytosis.

## Discussion

gH–gL is the main target for neutralizing MuHV-4 host entry and, as gH–gL is conserved, could potentially provide a general target for the *in vivo* neutralization of mammalian herpesviruses. Most gH–gL-directed herpesvirus neutralization is thought to block membrane fusion. However, this is based largely on the block being post-binding. gH–gL-directed MuHV-4 neutralization by mAbs T2C12 and 7D6 acted post-binding, but only prevented fusion indirectly; the immediate effect was to stop virions reaching the late endosomes where fusion normally occurs. This contrasted with gB-directed neutralization, which blocked membrane fusion and arrested virions in late endosomes. Thus, gH–gL-driven endocytosis provides a key neutralization target for MuHV-4, and potentially also for other herpesviruses that infect in similar ways.

Most analysis of the herpesvirus gH–gL has been done with HSV-1. Its gH–gL binds to integrins (Parry *et al.*, 2005) and has an essential role in fusion (Turner *et al.*, 1998). gH–gL-directed HSV-1 neutralization is thought to stop gH–gL interacting with gB (Chowdary *et al.*, 2010). This did not apply to MuHV-4, as its gB and gH are associated constitutively (Gillet & Stevenson, 2007). Therefore, the HSV-1 and MuHV-4 gH–gL may have different vulnerabilities. However, non-endocytic HSV-1 infection compresses several key events into the same site, making them difficult to distinguish, and the essential role of gL in HSV-1 gH maturation makes any subsequent roles that it may have difficult to define. In contrast, endocytic MuHV-4 infection makes some events anatomically distinct, and gH can reach virions without gL. Thus, the MuHV-4 and HSV-1 gH–gL may share functions that are simply more apparent with MuHV-4.

As with MuHV-4 and KSHV, the EBV gH–gL contributes to both epithelial-cell binding and membrane fusion; as with HSV-1, it binds to integrins (Chesnokova *et al.*, 2009). EBV-neutralizing mAbs to gH appear to disrupt gH–gL integrin binding or a post-binding conformation change (Chesnokova & Hutt-Fletcher, 2011). A block to integrin binding would have obvious parallels with the idea of mAb T2C12 blocking a gH–gL endocytic signal, and an EBV gH–gL conformation change would fit well with the MuHV-4 gH changing from gH–gL to gH-only after endocytosis (Gillet *et al.*, 2008b). We considered that mAb T2C12 might stabilize gH–gL against its conformation change. However, the similar phenotypes of T2C12-neutralized and gL^−^ virions made it more likely that neutralization blocked a ligand interaction that gL^−^ virions would constitutively lack. The endocytic signal was not delivered by gH–gL binding to HS, as this T2C12 does not block. Cell binding by recombinant gH–gL is entirely HS-dependent (Gillet *et al.*, 2008a), but other interactions could occur that depend on HS binding either for affinity or to alter the gH–gL conformation. This might explain why mAb 8F10 potently neutralized fibroblast and epithelial infections, despite only partially blocking cell binding.

MuHV-4 cell entry is probably most similar to that of KSHV, as MuHV-4 and KSHV are genetically closer to each other than either is to EBV. Whether KSHV neutralization blocks cell binding, endocytosis or membrane fusion is not well-defined. However, KSHV genome delivery to the nucleus depends on RhoA-dependent signalling (Chandran, 2010), and gH–gL appears to have a ligand besides HS (Hahn *et al.*, 2009) through which signalling could occur. gL^−^ bovine herpesvirus-4 also shows defective endocytic transport similar to that of gL^−^ MuHV-4 (Lété *et al.*, 2012). Therefore, pro-endocytic gH–gL signalling may be widespread among rhadinoviruses and an important component of their cell entry.

## Methods

### 

#### Cells.

NMuMG epithelial cells (ATCC CRL-1636), BHK-21 fibroblasts (ATCC CCL-10), Vero epithelial cells (ATCC CCL-81) and RAW-264 monocytes (ATCC TIB-71) were grown in Dulbecco’s modified Eagle’s medium with 2 mM glutamine, 100 U penicillin ml^−1^, 100 µg streptomycin ml^−1^ and 10 % FCS (complete medium).

#### Viruses.

The gL^−^ STOP mutant of MuHV-4 (Gillet *et al.*, 2007d), the lytically de-regulated M50 mutant (May *et al.*, 2004) and the EF1α–eGFP reporter virus (May & Stevenson, 2010) have been described previously. HSV-1 C12 (strain SC-16) expressing eGFP from a human cytomegalovirus IE1 promoter was kindly provided by Dr S. Efstathiou (Division of Virology, University of Cambridge, UK). MuHV-4 stocks were grown in BHK-21 cells (de Lima *et al.*, 2004). Cell debris was removed by low-speed centrifugation (1000 ***g***, 10 min) and virions were recovered from supernatants by high-speed centrifugation (38 000 ***g***, 90 min). The facts that virion preparations appeared as size-uniform dots and were negative for the post-endocytic MG-1A12 gB epitope (Gillet *et al.*, 2008c) confirmed that they were free of infected-cell debris. HSV-1 stocks were grown in Vero cells. The cells were lysed by freeze–thawing three times. Cell debris was removed by low-speed centrifugation (1900 ***g***, 10 min). MuHV-4 stocks were titrated by plaque assay on BHK-21 cells and HSV-1 stocks on Vero cells.

#### Antibodies.

MuHV-4-specific mAbs and immune serum were derived from MuHV-4-infected BALB/c mice. The mAbs used here were as follows: T2C12 (gH–gL, neutralizing, IgG_2a_), 7D6 (gH–gL, neutralizing, IgG_2a_), 8F10 (gH–gL, blocks HS binding, neutralizing, IgG_2a_), SC-9E8 (gB pre-fusion, neutralizing, IgG_2a_), SC-9A5 (gB pre-fusion, neutralizing, IgG_3_), BN-1A7 (gB pre-fusion, IgG_2a_), MG-1A12 (gB post-fusion, IgG_2a_), BN-8C3 (ORF75c, IgG_1_), BH-6H2 (gp150, IgG_2b_), BH-6D3 (ORF25, IgG_1_), 3F7 (gN, IgG_2a_). See also Table S1. Hybridoma supernatant was concentrated by ammonium sulfate precipitation, dialysed against PBS and quantified by Mancini assay (Mancini *et al.*, 1965). We also used rat anti-mouse LAMP-1 mAb (clone 1D4B; BD Biosciences) and rabbit anti-EEA-1 polyclonal antibody (pAb) (Abcam). The HSV-1 gD-specific mAb LP2 was kindly provided by Dr C. Crump (Division of Virology, University of Cambridge, UK). Direct labelling of mAb MG-1A12 with Alexa Fluor 488 was done with an APEX antibody labelling kit (Invitrogen).

#### Immunofluorescence.

NMuMG or Vero cells were seeded overnight onto glass coverslips. MuHV-4 or HSV-1 virions were bound to the cells at 4 °C (2 h). The cells were then washed three times in ice-cold PBS to remove unbound virions, and either fixed or first incubated for the indicated time at 37 °C in complete medium with or without neutralizing antibodies. After one wash in ice-cold PBS, fixation was achieved by adding ice-cold 4 % formaldehyde in PBS and leaving at room temperature for 30 min (mAbs BN-8C3, BH-6D3, BH-6H2, 3F7 and LP2) or 1 h (mAbs SC-9A5, BN-1A7 and MG-1A12). Fixation was stopped by adding 0.1 M glycine (15 min, room temperature), followed by three washes in PBS. The cells were then permeabilized with 0.1 % Triton X-100 (30 min, room temperature), blocked with 2 % BSA 0.1 % Tween 20 (overnight, 4 °C), stained with primary mAbs (1 h, room temperature), washed three times in PBS 0.1 % Tween 20, stained with secondary antibodies with 1 µg DAPI ml^−1^ (1 h, room temperature), washed three times in PBS 0.1 % Tween 20 and once in H_2_O, and mounted in ProLong Gold (Invitrogen). Secondary antibodies (goat anti-rat IgG, goat anti-rabbit IgG, and goat anti-mouse IgG, IgG_1_, IgG_2a_, IgG_2b_ or IgG_3_, labelled with Alexa Fluor 488 or 568) were all from Invitrogen. Images were acquired on Leica TCS SP2 and SP5 AOBS confocal laser-scanning microscopes with settings specific for DAPI (excitation, 405 nm; recording, 410–470 nm), Alexa Fluor 488 (excitation, 488 nm; recording, 493–550 nm) and Alexa Fluor 568 (excitation, 561 nm; recording, 566–700 nm). Images were analysed with ImageJ. Images show single *z* stacks, except in Fig. S1, where maximum-intensity projections from 10 *z* stacks spanning the entire depth of the cells are show. The thresholded Mander’s split co-localization coefficients and virion-particle counts were determined with ImageJ.

#### Flow cytometry.

MuHV-4-exposed cells were trypsinized and washed in PBS. Viral eGFP expression was then detected by flow cytometry using a FACSCalibur (BD Biosciences).

#### Cell ELISA.

NMuMG cells were seeded overnight into 96-well plates. MuHV-4 virions were bound at 4 °C (2 h). The cells were then washed three times in ice-cold PBS to remove unbound virions, and either fixed directly or first incubated at 37 °C in complete medium with or without neutralizing antibodies. After one wash in ice-cold PBS, cells were fixed by adding ice-cold 4 % formaldehyde in PBS and leaving at room temperature for 45 min (mAb BH-6D3) or 1 h (all other mAbs). Fixation was then stopped by incubation with 0.1 M glycine (15 min, room temperature), followed by three washes in PBS. The cells were then permeabilized with 0.1 % Triton X-100 (30 min, room temperature), blocked with 2 % BSA 0.1 % Tween 20 (overnight, 4 °C), then incubated with primary mAbs diluted in 2 % BSA 0.1 % Tween 20 (2 h, room temperature), followed by three washes in PBS 0.1 % Tween 20. The plates were then incubated with alkaline phosphatase-conjugated goat anti-mouse IgG_1_, IgG_2a_, IgG_2b_ and IgG_3_ pAb (Southern Biotech) diluted in 2 % BSA 0.1 % Tween 20 (2 h, room temperature), followed by six washes in PBS 0.1 % Tween 20. Bound secondary antibodies were detected by incubation with SIGMA*FAST* *p*-nitrophenyl phosphate substrate (Sigma-Aldrich) and reading *A*_405_ on a Benchmark Microplate Reader (Bio-Rad). For each antibody, the background was taken as the absorbance reading with uninfected cells and was subtracted from all values.
